# Virological Characterization of Pigs with Erythema Multiforme

**DOI:** 10.3390/microorganisms10030652

**Published:** 2022-03-18

**Authors:** Sabrina Halecker, Vasileios Papatsiros, Dimitra Psalla, Ludwig Krabben, Benedikt Kaufer, Joachim Denner

**Affiliations:** 1 Institute of Virology, Free University Berlin, 14163 Berlin, Germany; sabrina.halecker@fu-berlin.de (S.H.); ludwig.krabben@fu-berlin.de (L.K.); benedikt.kaufer@fu-berlin.de (B.K.); 2 Clinic of Medicine (Porcine Medicine), Faculty of Veterinary Medicine, University of Thessaly, GR 43100 Karditsa, Greece; vpapatsiros@vet.uth.gr; 3 Laboratory of Pathology, School of Veterinary Medicine, Aristotle University of Thessaloniki, GR 54124 Thessaloniki, Greece; dpsalla@vet.auth.gr

**Keywords:** porcine viruses, erythema multiforme, porcine endogenous retroviruses, porcine lymphotropic herpesviruses, porcine circoviruses, porcine cytomegalovirus

## Abstract

Erythema multiforme in pigs is an acute, self-limiting disease characterized by red skin areas and often associated with anorexia, fever and respiratory problems. The cause of the disease remains unknown. In a recent study, animals of a commercial breeding herd in Greece were examined, and all animals were found seropositive for porcine reproductive and respiratory syndrome virus (PRRSV). However, neither PRRSV and porcine circovirus type 2 (PCV2) viremia nor antibodies against Aujeszky’s disease virus, African swine fever virus and classical swine fever virus were detected. Here, an extended examination of these pigs was performed on a wide range of porcine viruses using highly sensitive polymerase chain reaction (PCR)-based methods. Affected skin of five animals revealed the presence of porcine lymphotropic herpesvirus-1 (PLHV-1) in all cases, PLHV-2 in one animal and PLHV-3 in four animals. However, neither porcine cytomegalovirus (PCMV) nor porcine circoviruses (PCV1, PCV2, PCV3 and PCV4) were detected. In blood samples, PLHV-1 was present in two animals and PLHV-2, PCV2 and PCV3 in one individual, with PCMV, PCV1 and PCV4 in none of the animals. In one animal, four viruses were found in the blood (PLHV-1, PLHV-2, PCV2 and PCV3). A PRRSV viremia was also not detected. All animals carried porcine endogenous retrovirus C (PERV-C) in their genome, but recombinant PERV-A/C was not detected. The results suggest that porcine viruses may be involved in erythema multiforme in these animals and that further studies are needed to assess the role of these pathogens in the disease.

## 1. Introduction

Erythema multiforme is a disease described not only in humans but also in pigs and other animals. In humans, erythema multiforme is characterized by skin eruption with typical target lesions. It is acute and self-limiting and usually resolves without complications. There may be mucous membrane involvement. Erythema multiforme in humans is considered to be a hyperergic mucocutaneous immune-mediated reaction to infections. Herpes simplex virus (HSV) is usually the causative agent in the majority of adults [1]. HSV causes mainly herpes labialis and, less frequently, genital herpes. In children, adolescents and young adults, a large percentage of erythema multiforme cases is caused by *Mycoplasma pneumoniae*, whereby the target lesions predominantly occur on the trunk. Other triggers of erythema multiforme may be parapoxvirus, varicella-zoster virus, adenoviruses, hepatitis viruses, human immunodeficiency virus and cytomegalovirus. Fungal infections have also been reported to be associated with erythema multiforme [2].

In pigs, skin lesions or skin abnormalities, such as abnormal color changes, are caused by bacterial pathogens (e.g., erysipelas, salmonellosis, pasteurellosis, pleuropneumonia and Glasser disease) or viral pathogens (e.g., classical swine fever, African swine fever and dermatitis/nephropathy syndrome due to porcine circovirus type 2 (PCV2) infections) [3,4,5]. However, septicemia or toxemia can cause erythema or cyanosis characterized by red to purple discoloration, especially on the extremities and easily seen in white pig breeds [3]. Erythema multiforme associated with respiratory disease was recently reported in sows of a Greek pig breed [6]. In that study, the animals were housed under stressful group-housing conditions, and a subclinical infection or an interaction of different respiratory pathogens (*Actinobacillus pleuropneumoniae*, *Haemophilus parasuis*, *Pasteurella multocida*, *Streptococcus suis*) seems to have been activated, negatively affecting the health status and performance of the breeding stock. All animals were found seropositive for porcine reproductive and respiratory syndrome virus (PRRSV); however, no PRRSV, porcine circovirus type 2 (PCV2) viremia, antibodies against Aujeszky’s disease virus (suid herpesvirus 1, SuHV-1), African swine fever virus or classical swine fever virus were detected [6]. Moreover, cases of erythema multiforme have been reported in dogs after canine parvovirus-2 infection [7] and in wild animals, such as ferrets and spotted hyenas [8,9]. In Göttingen minipigs, a syndrome related to erythema multiforme was described, called “Dippity Pig Syndrome”. Red streaks on the back of the pigs develop rapidly. Soon, the animals start oozing, and the pig arches its back and may vocalize in pain [6,10].

To analyze whether virus infections may be associated with erythema multiforme as described in humans, diseased pigs in a pig farm in Greece were screened by molecular biological methods, including highly sensitive PCR-based methods. These methods had been previously developed to screen donor pigs and recipients before and after xenotransplantation [11,12,13].

## 2. Materials and Methods

### 2.1. Animals

The animals analyzed in the present case report were part of a commercial breeding stock (Large White × Landrace), suffering from a disease that was described previously and characterized as erythema multiforme associated with respiratory disease [6]. The animals studied here were the granddaughters of the previously studied animal. The five selected pregnant sows (Table 1) were in group-housing rooms during the dry-period stage. They lived in the same building but in three different pens with courtyard space.

The capacity of the farm, which is located in Central Greece, was 620 sows under production. All sows were moved to dry period after their weaning for the application of artificial insemination, remained for 30–35 days in individual stalls and then were removed to group-housing rooms. All sows of the farm were vaccinated against PRRSV; SuHV-1; porcine parvovirus 1; atrophic rhinitis, caused by infection with toxigenic *Pasteurella multocida*; erysipelas, caused by infection with *Erysipelothrix rhusiopathiae*, *Escherichia coli* and *Clostridium perfringens*. Weaners were vaccinated against PCV2 and *Mycoplasma hyopneumoniae*. Suvaxyn PRRS MLV (ZOETIS) was used against PRRSV. For antiparasitic control, all breeding females were treated with a single ivermectin injection 14 days prior to each farrowing. The feed provided to the animals was self-prepared based on a corn/barley/wheat–soya feed ration, depending on the season.

**Figure 1:**
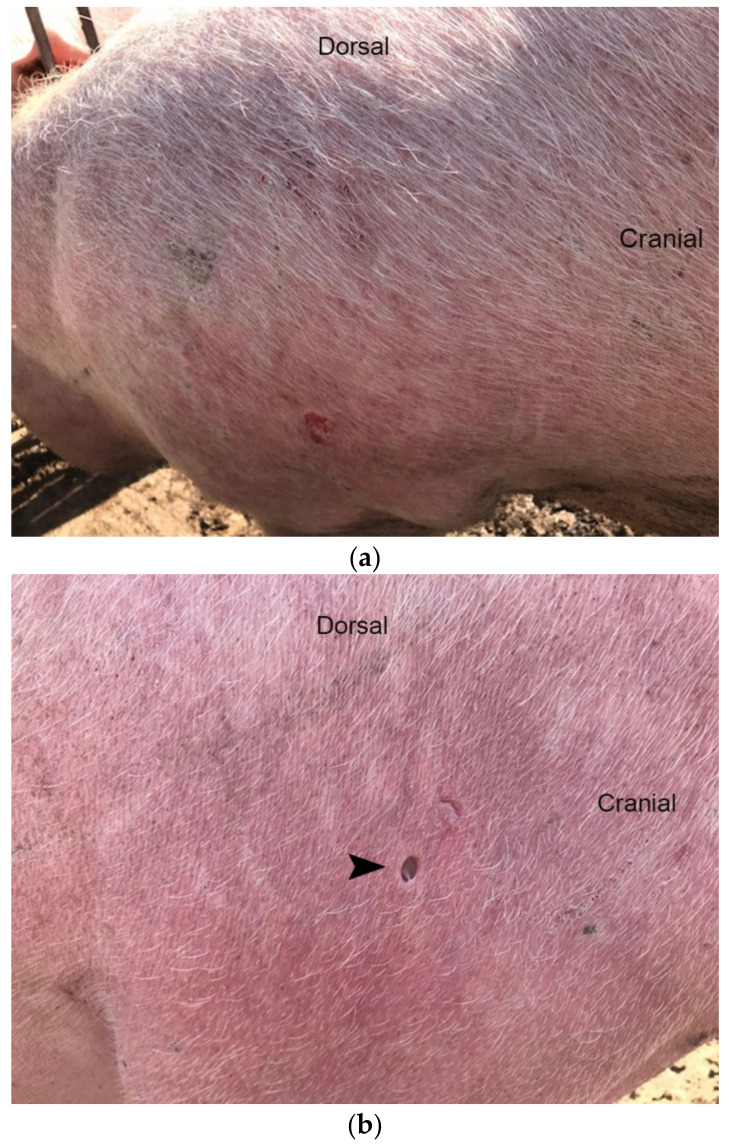
(**a**) Hypotrichosis, erythema and occasional erosions (picture from lateral abdomen–hip region). (**b**) Location of skin sampling (arrowhead) for histological examination of a sow with erythema multiforme.

The selected sows showed clinical signs of previously described erythema multiforme associated with respiratory disease [6]. Particularly, the affected skin areas were red-colored, raised lesions that were noticeable on the whole body of the sows, but the neck and face seem to be more affected (Figure 1a,b). Moreover, the diseased sows showed depression, decreased appetite, pyrexia (40–41.5 °C), stiffness and respiratory signs with moderate dyspnea, eye and nasal discharge. In severely diseased sows, mucous to bloody ocular and nasal excretions were noticed.

During an outbreak of these clinical signs, the percentage of diseased animals ranged from 50 to 60%. The mean number of each batch of sows/gilts in dry period is about 220–250 animals.

### 2.2. Ethics Statement

All procedures were conducted according to the ethical standards in the Helsinki Declaration of 1975, as revised in 2000, as well as the national law and after receiving approval (approval number 104/16.11.2021) from the Thessaly University Ethics Committee. 

### 2.3. Sample Collection and Histology

Blood and skin punches from the affected skin areas of five pregnant sows suffering from red-colored, raised skin lesions were collected [14], frozen and delivered to the Institute of Virology at Freie Universität Berlin for virological investigations. 

For histological examinations, one punch biopsy (6 mm in diameter) was taken per animal. The biopsy was fixed in 10% buffered formalin, embedded in paraffin, cut and stained with hematoxylin and eosin following standard procedures [15].

### 2.4. DNA Extraction

DNA extraction from frozen blood was set up by using a DNeasy blood and tissue kit (Qiagen, Hilden, Germany) according to the manufacturer’s recommendations. The skin samples were treated prior to DNA extraction with collagenase (Sigma-Aldrich, St. Louis, MO, USA, final concentration 125 U/mL) and hyaluronidase (Sigma-Aldrich, St. Louis, MO, USA, final concentration 100 U/mL) and incubated at 37 °C at 500 rpm for 30 min on a thermoshaker (Eppendorf, Hamburg, Germany), followed by 5 min centrifugation at 1000× *g* at room temperature. DNA was extracted from the pellet using the DNeasy blood and tissue kit. To quantify DNA, a NanoDrop ND-1000 spectrophotometer (Peqlab Biotechnologie GmbH, Erlangen, Germany) was used, and the samples were stored at −20 °C until further processing.

### 2.5. PERV Testing

Briefly, to detect PERV-C, a quantitative TaqMan-PCR (real-time PCR) was performed as described by Takeuchi et al. [16]. The species-specific primers are located at the envelope gene of PERV-C (PERV envC). A SensiFAST Probe No-ROX one-step kit (Meridian Bioscience, Cincinnati, OH, USA) was used for the master mix. PCRs were run on a qTOWER^3^ G (Analytik Jena, Jena, Germany) with a final volume of 20 μL.

The investigation of PERV-A/C was performed as a conventional PCR based on a primer-probe mix detecting a 1.266 basepair amplicon as described by Wood et al. [17]. For this PCR, AmpliTaq DNA polymerase (Applied Biosystems, Waltham, MA, USA) was used in a PCR reaction volume of 20 μL plus 5 μL DNA template. The PCR was carried out on a Biometra TRIO cycler (Analytik Jena, Jena, Germany).

### 2.6. Testing of PCMV, PLHV and PCV

For the molecular biological investigation of several porcine viruses (PCMV, PLHV-1, PLHV-2, PLHV-3, PCV1, PCV2, PCV3 and PCV4), species-specific real-time PCR methods were applied as previously described (Table 2) [18,19,20,21,22]. The PCR conditions concerning the detection of PCMV, PLHV-1, PLHV-2, PCV1, PCV2, PCV3 and PCV4 were recently published by Halecker et al. [11]. The detection of PLHV-3 was adapted, and a real-time PCR approach was applied, as described by McMahon et al. [20]. The master mix for all real-time PCRs was prepared using a SensiFAST Probe No-ROX kit (Meridian Bioscience, Cincinnati, OH, USA). Primers and probes located at glycoprotein B were used for the detection of PLHV-3. Simultaneously, porcine glyceraldehyde-3-phosphate dehydrogenase (pGAPDH) was detected in all samples to verify correct sample preparation [23]. Gene-block gene fragments (Integrated DNA Technologies, IDT, Coralville, IA, USA) containing the corresponding sequence of the gene of interest served as positive control [11]. For the amplification of PLHV-3 amplicons, the temperature–time conditions started with an activation step at 90 °C for 10 min, followed by 45 cycles comprising a denaturation step at 90 °C for 30 s and a merged annealing-extension step at 59 °C for 30 s. All real-time PCRs were run on a qTOWER^3^ G (Analytik Jena, Jena, Germany).

### 2.7. PRRSV Testing 

PRRSV testing was performed by the private laboratory “VET IN PROGRESS, GREECE”. RNA was extracted using a QIAamp cador pathogen mini kit (INDICAL BIOSCIENCE GmbH, Leipzig, Germany) according to the manufacturer’s instructions. Carrier RNA was added to each sample, as it enhances the adsorption of viral RNA to the silica membranes. An internal positive control RNA (RNA IPC Target; Ingenetix) was added to all samples to ensure recovery of RNA and prove the functionality of the PCR reaction. An RT-PCR kit (ViroReal Kit PRRS Virus EU & NA 1.1; Ingenetix GmbH, Vienna, Austria) was used for the detection of both viral lineages of the PRRSV, PRRSV-1 (European) and PRRSV-2 (North American) strains. The specific gene targets of the commercial assay are proprietary and not publicly available. The reaction conditions involved a reverse-transcription (RT) step at 55 °C for 10 min and RT inactivation and initial denaturation step at 95 °C for 1 min, followed by 45 cycles of a denaturation step at 95 °C for 10 s and a combined annealing-extension step at 60 °C for 1 min. A Roche LightCycler 2.0 was used.

### 2.8. Treatment 

The five animals analyzed here were treated twice intramuscularly with amoxicillin (Animox LA, Univet LTD, Tullyvin, Cootehill, County Cavan, Ireland) at dose of 15 mg/kg body weight, with an interval of 48 h between treatments. However, the treatment outcomes were not adequate. 

## 3. Results

### 3.1. Diseased Animals and Histology of the Affected Skin

Typical lesions were detected in all examined sows (Figure 1a,b), and the intensity was similar. These clinical signs were observed in diseased animals, especially in periods when stress factors, e.g., after artificial insemination, movement of sows from the dry-period room to the farrowing room 3–7 days before farrowing. The selected animals were characterized by very severe and obvious skin lesions. 

**Figure 2:**
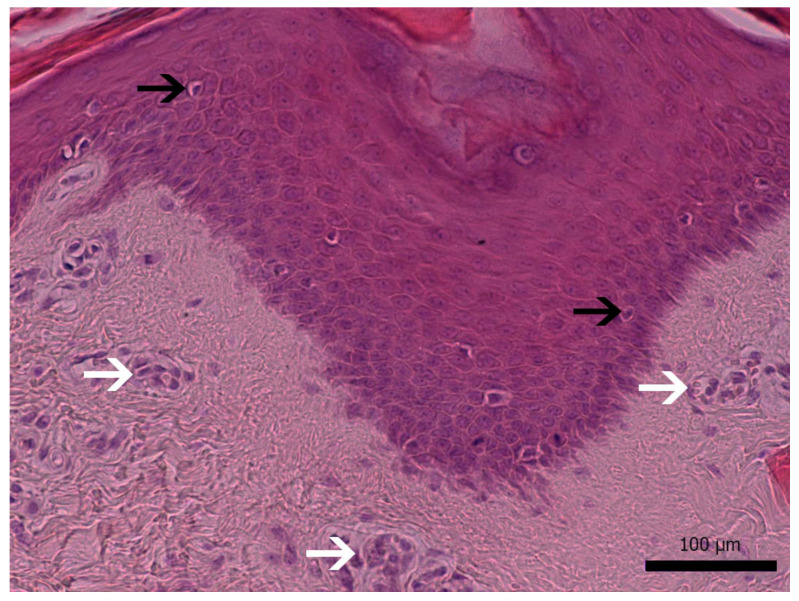
Histopathological findings: severe epidermal hyperplasia with scattered apoptotic bodies (black arrows). Prominent capillaries on superficial dermis (white arrows). H&E stain, magnification ×200 (bar: 100 μm).

Histopathological examination of skin samples revealed hyperkeratotic and rare parakeratotic hyperkeratosis and moderate-to-severe epidermal hyperplasia. Scattered apoptotic bodies and few lymphocytes were present in all layers of the epidermis and follicular infundibular epithelium. Superficial and middle dermis presented increased vascularization and hyperemia (Figure 2).

### 3.2. Results of Virus Screening

DNA was isolated and screened for several viruses using PCR or real-time PCR (Table 3). PCMV, PCV1, PCV4, PRRSV-1 and PRRSV-2 were detected neither in the skin nor in the blood. PLHV-1 was the virus with the highest detection rate. It was found in all affected skin samples and in two of five blood samples. PLHV-2 was identified in the affected skin and in the blood of one animal. PLHV-3 was found in four of the animals but only in the affected skin and not in the blood. Intriguingly, PLHV-3 was not detected in pig #4, which was the only pig infected with PLHV-2. Porcine circoviruses were only found in one pig (pig #4), which was positive for both PCV2 and PCV3. Therefore, four viruses were found in a single animal (pig #4): PLHV-1, PLHV-2, PCV2 and PCV3.

PERV-A and PERV-B are present in the genome of all pigs, whereas PERV-C is not. PERV-C was found in all five pigs, although with different Ct values, suggesting that the copy number might vary in these pigs. Since PERV-C is present, recombination with PERV-A resulting in a PERV-A/C can occur. However, our analysis revealed that no recombination took place. 

In our previous publication, the animals suffering from erythema multiforme were all seropositive for PRRSV but PCR negative for this virus [6]. The animals studied here were their granddaughters and were also negative in PCR testing for both PRRSV strains, PRRSV-1 and PRRSV-2. Since in both cases, the animals were vaccinated against PRRSV, the measured immune response was due to the vaccine; therefore, it is unlikely that PRRSV is involved in the pathogenesis of erythema multiforme.

## 4. Discussion

Erythema multiforme is a multifactorial disease described for humans [1,24,25], pigs [3,4,5,6], dogs [7] and wild animals, such as ferrets and spotted hyenas [8,9]. The most studied risk factors are drugs, bacterial, viral and fungal infections and/or stress. In humans, erythema multiforme with characteristic skin lesions was observed following anti-tumor necrosis factor (TNF)-α medication with adalimumab for rheumatoid arthritis [26] or after exposure to different herbicides [27]. Pediatric erythema multiforme was most commonly attributed to upper respiratory and *Mycoplasma pneumoniae* infections, whereas adult erythema multiforme was mainly assigned to HSV infections [28]. Since the SARS-CoV-2 pandemic, numerous cases of erythema multiforme in infected patients have been reported [23,24,29]. Erythema multiforme cases were reported after vaccination against the coronavirus, as well as after other vaccinations [30,31,32]. 

In pigs, group housing can result in fighting, fear and anxiety coinciding with the onset of disease [33]. Therefore, stress is another risk factor associated with erythema multiforme [6,10]. 

The fact that several viruses were detected in the analyzed pigs suggests that they may contribute to the diagnosed erythema multiforme, possibly together with stress or other cofactors. However additional investigations are required to prove this. In the affected skin, PLHV-1 was present in all five animals. Only two of the five animals were positive for PLHV-1 in the blood, suggesting that virus replication occurs mostly in the skin and is not systemic. As a lymphotropic herpesvirus, PLHV-1 certainly replicates in lymphocytes in the skin. Until now, no association between PLHVs and any pig diseases had been described. PLHV-1 causes a post-transplantation lymphoproliferative disorder (PTLD) after experimental allogenic bone marrow transplantations in minipigs [34,35,36]. This disorder is similar to human PTLD, a serious complication of solid human organ transplantation linked to Epstein–Barr virus (EBV). Despite their high prevalence of up to 80% in some countries, the relevance of PLHVs for the swine industry appears to be low. Their transmission occurs mainly horizontally, but vertical transmission is possible. Although some genetically modified pigs generated for xenotransplantation trials were found to be positive for PLHV-1, it was not transmitted to the non-human primate recipients (for review, see [37]). 

Interestingly, in one animal, pig #4, four viruses were found: PLHV-1, PLHV-2, PCV2 and PCV3. PCV2 was found despite vaccination against the virus. PCV1 and PCV4 were not found. PCMV, a roseolovirus closely related to human herpesviruses HHV-6 and HHV-7, was also not found in any of the analyzed animals. PCMV is widely distributed in pigs, and sensitive detection methods were developed because PCMV poses a serious risk for xenotransplantation. Transmission of PCMV with transplanted pig hearts drastically reduced the lifetime of the xenotransplant in baboons [38] and of pig kidneys in rhesus monkeys and baboons (for review, see [39]). 

The methods used here to detect different viruses are highly sensitive and specific [12]; however, there are other methods that can be used to screen for viruses, e.g., microarray investigations or next-generation sequencing. At the moment, such microarrays to detect porcine viruses and powerful bioinformatic tools concerning pig viruses are not available. However, in future studies, such approaches should be used to obtain more information about the viruses present in the diseased pig or the affected skin area.

PERVs are present in the genome of pigs. PERV-A and PERV-B are present in the genome of all pigs. They can be released as infectious virus particles and infect human cells in vitro and therefore pose a risk for xenotransplantation [40]. PERV-C is an ecotropic virus infecting only pig cells, and it is not present in all pigs. PERV-C was found as integrated proviruses in the genome of all five analyzed animals (Table 3). PERV-A and PERV-C can recombine in living pigs; the recombinant PERV-A/C is able to infect human cells and is characterized by high replication rates (for review, see [41]). PERV-A/C was not found in these animals with erythema multiforme (Table 3). In previous studies, the copy number of the integrated proviruses differed depending on the pig breed, the animal and the organ tested (for review, see [42]). PERVs are active and able to infect new cells in the living animal and to integrate de novo. This explains varying copy numbers in different organs of a single animal. Expression of PERV was not analyzed in this study. Although PERV was found to be produced as virus particles by pig lymphoma cells and was highly expressed as mRNA in pig melanomas, there is no evidence connecting PERVs with a pig disease (for review, see [43]). 

## 5. Conclusions

Five sows with diagnosed erythema multiforme were analyzed using sensitive PCR-based methods. PLHV-1 was found in all animals, and PLHV-2, PCV2 and PCV3 were found in some of the animals, but no PCMV, PCV1, PCV4, PRRSV-1 or PRRSV-2 was detected. This study indicates that viruses may be involved in the pathogenesis of the disease; however, this has to be proven by additional investigations of larger numbers of animals and using additional methods. 

## Figures and Tables

**Table 1:** Data on the individual animals of the Greek breed tested in this study.

Animal ID	Parity	Age	Animal Status
1	3	23.5 months	breeding sow
2	4	26 months	breeding sow
3	2	18.5 months	breeding sow
4	3	23 months	breeding sow
5	1	14 months	breeding sow

**Table 2:** Primers and probes used in this study.

PCR Assay	Primer/Probe	Sequence 5’-3’	Reference
PCMV	PCMV-Fwd	ACT TCG TCG CAG CTC ATC TGA	Mueller et al. [18], modified
	PCMV-Rev	GTT CTG GGA TTC CGA GGT TG	
	PCMV-Probe	6FAM-CAG GGC GGC GGT CGA GCT C-BHQ	
PLHV-1	PLHV-1 (1125)-Fwd	CTC ACC TCC AAA TAC AGC GA	Chmielewicz et al. [19]
	PLHV-1 (1125)-Rev	GCT TGA ATC GTG TGT TCC ATA G	
	PLHV-1 (1125)-Probe	6FAM-CTG GTC TAC TGA ATC GCC GCT AAC AG-TAMRA	
PLHV-2	PLHV-2 (1155)-Fwd	GTC ACC TGC AAA TAC ACA GG	Chmielewicz et al. [19]
	PLHV-2 (1155)-Rev	GGC TTG AAT CGT ATG TTC CAT AT	
	PLHV-2 (1155)-Probe	6FAM-CTG GTC TAC TGA AGC GCT GCC AAT AG-TAMRA	
PLVH-3	PLHV-3 (210s)-Fwd	AAC AGC GCC AGA AAA AAA GG	McMahon et al. [20]
	PLHV-3 (210as)-Rev	GGA AAG GTA GAA GGT GAA CCA TAA AA	
	PLHV-3 (210)-Probe	6-FAM CCA AAG AGG AAA ATC-MGB	
PCV1	PCV1 (F2020)-Fwd	AAC CCC ATA AGA GGT GGG TGT T	Chen et al. [21], modified
	PCV1 (F2020)-Rev	TTC TAC CCT CTT CCA AAC CTT CCT	
	PCV1 (F2020)-Probe	6FAM-TCC GAG GAG GAG AAA AAC AAA ATA CGGGA-BHQ1	
PCV2	PCV2 (F2020)-Fwd	CTG AGT CTT TTT TAT CAC TTC GTA ATG GT	Chen et al. [21], modified
	PCV2 (F2020)-Rev	ACT GCG TTC GAA AAC AGT ATA TAC GA	
	PCV2 (F2020)-Probe	6FAM-TTA AGT GGG GGG TCT TTA AGA TTA AAT TCT CTG AAT TGT-TAMRA	
PCV3	PCV3-Fwd	AGT GCT CCC CAT TGA ACG	Palinski et al. [22]
	PCV3-Rev	ACA CAG CCG TTA CTT CAC	
	PCV3-Probe	6FAM-ACC CCA TGG CTC AAC ACA TAT GAC C-BHQ1	
PCV4	PCV4 (F2020)-Fwd	ATT ATT AAA CAG ACT TTA TTT GTG TCA TCA CTT	Chen et al. [21]
	PCV4 (F2020)-Rev	ACA GGG ATA ATG CGT AGT GAT CAC T	
	PCV4 (F2020)-Probe	6FAM-ATA CTA CAC TTG ATC TTA GCC AAA AGG CTC GTT GA-BHQ1	
PERV-C	PERV envC-Fwd	CTGACCTGGATTAGAACTGG	Takeuchi et al. [16]
	PERV envC-Rev	ATGTTAGAGGATGGTCCTGG	
	PERV envC-Probe	6FAM-CTC TAA CAT AAC TTC TGG ATC AGA CCC-BHQ1	
PERV-A/C	PERV-A env VRBF-Fwd	CCT ACC AGT TAT AAT CAA TTT AAT TAT GGC	Wood et al. [17]
	PERV-C env TMR-Rev	CTC AAA CCA CCC TTG AGT AGT TTC C	
pGAPDH	pGAPDH-Fwd	ACA TGG CCT CCA AGG AGT AAG A	Duvigneau et al. [23]
	pGAPDH-Rev	GAT CGA GTT GGG GCT GTG ACT	
	pGAPDH-Probe	HEX-CCA CCA ACC CCA GCA AGA GCA CGC-BHQ1	

**Table 3:** Screening for porcine viruses in pigs with erythema multiforme.

Material Tested	Animal ID		Ct Values										
		PCMV	PLHV-1	PLHV-2	PLHV-3	PCV1	PCV2	PCV3	PCV4	PRRSV-1	PRRSV-2	PERV-C	PERV-A/C
Skin	1	No Ct	33.50	No Ct	32.70	No Ct	No Ct	No Ct	No Ct	No Ct	No Ct	13.68	-
samples	2	No Ct	31.87	No Ct	32.95	No Ct	No Ct	No Ct	No Ct	No Ct	No Ct	14.56	-
	3	No Ct	33.63	No Ct	31.28	No Ct	No Ct	No Ct	No Ct	No Ct	No Ct	14.38	-
	4	No Ct	31.82	33.43	No Ct	No Ct	No Ct	No Ct	No Ct	No Ct	No Ct	15.17	-
	5	No Ct	32.48	No Ct	37.36	No Ct	No Ct	No Ct	No Ct	No Ct	No Ct	14.83	-
Blood	1	No Ct	32.84	No Ct	No Ct	No Ct	No Ct	No Ct	No Ct	No Ct	No Ct	16.00	-
	2	No Ct	No Ct	No Ct	No Ct	No Ct	No Ct	No Ct	No Ct	No Ct	No Ct	21.08	-
	3	No Ct	No Ct	No Ct	No Ct	No Ct	No Ct	No Ct	No Ct	No Ct	No Ct	19.44	-
	4	No Ct	31.62	31.43	No Ct	No Ct	37.33	39.22	No Ct	No Ct	No Ct	17.79	-
	5	No Ct	No Ct	No Ct	No Ct	No Ct	No Ct	No Ct	No Ct	No Ct	No Ct	nt	-

Ct = cycle threshold (indicates a negative result detected by a real-time PCR); nt = not tested; - (minus) = negative result detected by a conventional PCR.

## Data Availability

The data presented in this study are available in the article.
